# Deleterious impact of obstructive sleep apnea on autonomic nervous system control during rapid‐eye‐movement sleep in adult type 1 diabetes

**DOI:** 10.14814/phy2.70951

**Published:** 2026-06-16

**Authors:** Marion Faivre, Fares Gouzi, Bronia Ayoub, Justine Myzia, Orianne Villard, Amandine Suc, Maurice Hayot, François Roubille, Yves Dauvilliers, Jérôme Thireau, François Bughin

**Affiliations:** ^1^ University of Montpellier, PhyMedExp, CNRS, INSERM Montpellier France; ^2^ University Hospital Center of Montpellier Montpellier France; ^3^ Department of Endocrinology and Diabetology Montpellier University Hospital Montpellier France; ^4^ Intensive Care Unit, Cardiology Department University Hospital of Montpellier Montpellier France; ^5^ Sleep‐Wake Disorders Unit, Department of Neurology Gui‐de‐Chauliac Hospital, CHU Montpellier Montpellier France

**Keywords:** autonomic dysfunction, heart rate variability, sleep‐disordered breathing, type 1 diabetes

## Abstract

Obstructive sleep apnea (OSA) with an apnea‐hypopnea index (AHI) ≥ 15/h increases the cardiovascular disease (CVD) risk in type 1 diabetes (T1D). Cardiovascular autonomic neuropathy (CAN) is a key driver of CVD in diabetes and could be increased by autonomic nervous system (ANS) dysfunction related to OSA. We compared ANS control in T1D patients with AHI ≥ 15/h versus <15/h. T1D patients underwent polysomnography with heart rate variability analysis, cardiovascular reflex tests (CARTs), sudomotor function test, and urinary catecholamine analysis. Compared with patients with AHI < 15/h (*n* = 14), those with AHI ≥ 15/h (*n* = 7) showed a lower HFnu during REM sleep (12 ± 11 vs. 25 ± 14 nu, *p* < 0.05) and a higher heart rate (73 ± 8 vs. 64 ± 9 bpm; *p* < 0.05). A significantly higher urinary norepinephrine/epinephrine ratio was observed in patients with AHI ≥ 15/h. No significant group differences were observed regarding CAN. However, AHI correlated with systolic blood pressure at orthostatism (*r* = 0.54, *p* < 0.05). In T1D patients, OSA with AHI ≥ 15/h showed a lower parasympathetic activity and a sympathetic activation during sleep without any significant diurnal CAN proportion increase in patients with AHI ≥ 15/h. The potential impact of OSA on CAN and CVD risk in T1D patients should be assessed in further studies.

## 1 INTRODUCTION

Type 1 diabetes (T1D) causes macrovascular and microvascular complications leading to cardiovascular diseases (CVD). The relative risk of CVD in T1D patients is 10 times higher than in the general population (de Ferranti et al., 2014) and CVD events occur 10–15 years earlier than in matched non‐diabetic subjects (Soedamah‐Muthu et al., 2006). Moreover, after 60 years old, CVD risk is higher in patients with T1D compared to patients with type 2 diabetes (Patsoukaki et al., 2025).

Currently, most of these CVD determinants remain to be identified in T1D. Indeed, even in patients who achieve a strict glycemic control at target, the risk of CVD remains approximately twice as high (Lind et al., 2014). Among potential cardiovascular factors in well‐controlled T1D patients, obstructive sleep apnea (OSA)—a treatable chronic disease caused by a recurrent upper airway collapse during sleep—appears as a relevant candidate. Indeed, in a French cohort, moderate OSA patients (apnea‐hypopnea index (AHI) ≥ 15/h), which represent up to 40% of the T1D population, had a 3.96‐fold increased risk of macrovascular complications (Pépin et al., 2023).

One potential mechanistic link between OSA and cardiovascular complications in patients with T1D is cardiovascular autonomic neuropathy (CAN). Indeed, CAN is an autonomic nervous system dysfunction, resulting in abnormalities in heart rhythm control and vascular dynamics. It is assessed by cardiovascular autonomic reflex tests (CARTs) (Ewing et al., 1985; Tesfaye et al., 2010) and/or heart rate variability analyses (HRV) (Ziegler, 1994). In T1D without OSA, patients with CAN have an increased risk of cardiovascular disease and all‐cause mortality versus patients without CAN (Chowdhury et al., 2021). Thus, systematic screening for CAN has been recommended by the American Diabetes Association (Pop‐Busui et al., 2017).

In OSA patients without diabetes, sleep apnea induces an autonomic nervous system dysfunction during nocturnal periods. Using HRV assessment, the LF/HF ratio, a marker of sympatho‐vagal balance, has been correlated with the AHI, and this nocturnal increase in LF/HF ratio occurred in OSA patients with an AHI > 15/h (Palma et al., 2015; Park et al., 2008). Moreover, besides acute autonomic system changes related to OSA, there is large evidence of chronic autonomic nervous system dysfunction mimicking CAN in OSA patients. Studies have shown an increase in baseline daytime sympathetic activity and abnormal vagal reflex responses (Lombardi et al., 2019). Altogether, in the context of T1D, we hypothesized that OSA could constitute an aggravating factor for the diurnal and nocturnal autonomic control of heart rhythm. Yet, no study has ever compared diurnal and nocturnal markers of CAN between patients with an AHI< and ≥15/h, considering sleep architecture, specifically in T1D patients.

Therefore, given the higher risk of cardiovascular complications (Pépin et al., 2023) as well as the higher LF/HF ratio in OSA patients with an AHI ≥ 15 h, we aimed to compare in T1D patients the heart rate variability index LF/HF ratio during wakefulness and sleep stages between patients with sleep apnea syndrome with AHI ≥ 15/h and patients with AHI < 15/h. In addition, we compared between these groups other HRV indices during wakefulness such as cardiovascular autonomic reflex tests, sudomotor function, and urinary catecholamines.

## 2 MATERIALS AND METHODS

### 2.1 Study population

Patients with type 1 diabetes aged between 18 and 60 years old with a disease duration of at least 5 years were included, from regular follow‐up in the department of Endocrinology and Diabetology, Montpellier University Hospital. Patients were not included if they were treated by continuous airway pressure (CPAP), had chronic alcoholism, neuromuscular disease, drugs interfering with sinus variability (betablockers, antiarrhythmics, and ivabradine) or a pacemaker, or if they were pregnant. Written consent was obtained. The study was approved by review boards (CPP IDF1 n°2017‐A03438‐45), conducted in accordance with Helsinki's declaration and identified as NCT03605329 https://clinicaltrials.gov/study/NCT03605329.

During the first medical visit, patients gave informed consent and were included in the study. Patients data from their records have been extracted to gather information on their medical history, treatments including insulin therapy (pump or multi‐injections), and diabetes‐related complications (retinopathy, chronic kidney disease, macrovascular complications, peripheral neuropathy, and autonomic neuropathy). Then, patients performed diurnal functional assessments (Sudoscan®, CARTs), and on the following night, they underwent polysomnography (PSG). Fasting blood and urine samples were collected the next morning at 8 a.m.

### 2.2 Polysomnographic study

A polysomnographic study was conducted at the hospital using the Sleepware G3 system (Philips). Electroencephalography was recorded using six pairs of leads (2 occipital, 2 frontal, and 2 central) with two pairs of electro‐oculographic leads. Electromyographic leads were attached to the tibialis anterior and submentalis muscles. Airflow was continuously monitored using a thermistor and a nasal pressure cannula. Respiratory motions were tracked with the use of inductive plethysmography belts wrapped tightly around both the abdomen and chest. Sleep stages and events were evaluated and scored based on the guidelines established by the American Academy of Sleep Medicine (Berry et al., 2017). The details can be found in the Appendix S1. Patients were classified into none or mild OSA (AHI < 15) and moderate or severe OSA (AHI ≥ 15) groups. We also analyzed AHI during REM sleep (AHI REM) and during NREM sleep (AHI NREM, including N2 and N3 stages).

### 2.3 Autonomic system function assessments

#### 2.3.1 Heart rat variability

Short‐term HRV was analyzed over 5‐min period, in order to minimize the impact of micro‐arousals on sympathetic activity, as previously validated (Task Force of the ESC and NASPE, 1996). Both frequency‐domain and time‐domain analysis were performed. Five‐minute periods were manually extracted from PSG throughout each sleep stage (N2, N3, REM) and the wakefulness period right before sleep. One period was extracted for each sleep stage and for wakefulness. For sleep stages, the period was selected immediately before a sleep stage change, excluding the 5 min preceding the change and any segments containing arousals (Bonnet & Arand, 1997). If an arousal occurred, the 5 min prior to the arousal were extracted instead. For wakefulness period, the segment immediately before lights‐off was extracted. Sleep stages were scored manually, in accordance with the AASM guidelines. For N2 and N3 sleep stages, periods were selected during the first sleep cycle. For REM sleep, periods were selected during the last sleep cycle. Ectopic beats and artifacts were discarded before performing HRV analysis (Thireau et al., 2008, 2017), which was performed using Heart Rate Variability Analysis Software (v1.2, ADInstruments) (Pichot et al., 2016). The following time‐domain HRV parameters were analyzed: the standard deviation of all normal RR intervals (SDNN) and the root mean square successive differences in normal heart period series (RMSSD). The frequency‐domain indices were measured by using the fast Fourier transform according to different frequency ranges. They commonly include the total density of power spectrum (TP), the spectral densities of very low‐frequency band (VLF; ≤0.04 Hz), of low‐frequency band (LF; 0.04–0.15 Hz) and of high‐frequency band power (HF; 0.15–0.40 Hz) (Task Force of the ESC and NASPE, 1996). The others derivative parameters used were LFnu and HFnu (LFnu = LF/(TP‐VLF) × 100) and (HFnu = HF/(TP‐VLF) × 100) in which were low frequency and high frequency powers of heart rate variability expressed in normalized units (nu), and the LF/HF ratio.

#### 2.3.2 Cardiovascular autonomic reflex tests

Heart rate and blood pressure responses were assessed during 5 standardized maneuvers: heart rate response to deep breathing, heart rate response to standing, Valsalva maneuver, blood pressure response to standing up, and blood pressure response to sustained handgrip. The details and thresholds chosen (Chowdhury et al., 2021) can be found in the Appendix S1. Presence of CAN was assessed as follows: no CAN if all tests were normal, possible CAN if one test was abnormal, confirmed CAN if two tests were abnormal, and severe CAN if two or more tests were abnormal and associated with orthostatic hypotension (Tesfaye et al., 2010), defined as a decrease in systolic blood pressure of 20 mmHg or more.

#### 2.3.3 Sudomotor function

Sudomotor function was assessed using Sudoscan® (Impeto Medical, Paris, France), according to the validated methodology (Selvarajah et al., 2015). Briefly, patients were asked to place their hands and feet on large electrodes for 2 min, during which time a low‐voltage current was applied, stimulating the sympathetic sudomotor fibers. Measurement is based on an electrochemical reaction between electrodes and chloride ions, released by sweat glands. The flow of chloride‐dependent current is expressed as electrochemical skin conductance (ESC) for hand and feet, expressed in microSiemens (μS). An ESC below 60 μS was considered abnormal (Casellini et al., 2013).

#### 2.3.4 Blood and urinary biomarkers

Catecholamines (epinephrine (E), norepinephrine (NE), normetanephrine, and metanephrine) were measured from a urinary sample collected in the morning upon awakening, using high performance liquid chromatography (HPLC, Electrochimie Waters) and data were normalized to urinary creatinine. NE/E ratio was calculated to assess peripheral sympathetic nervous system activity (Robertson et al., 1979; Yamaguchi et al., 1988). From this urinary sample, albuminuria was measured to screen for diabetic nephropathy through assessment of microalbuminuria (mg/mmol of creatinine).

Fasting blood samples were collected to analyze glycosylated hemoglobin (HbA1c, %), creatinine levels (mg/dL), estimated glomerular filtration rate (eGFR, mL/min.1.73m^2^), using high performance liquid chromatography. Venous blood samples were collected from the antecubital vein using EDTA tubes, heparinized tubes, or dry tubes. The samples were centrifuged at 4°C.

### 2.4 Statistical analysis

Descriptive statistics were calculated for all variables. Normal distribution was assessed by Kolmogorov–Smirnov test. Data are presented as mean ± standard deviation or number (percentage). Heart rate, R‐R intervals, LFnu and HFnu powers were analyzed using raw data, whereas RMSSD, SDNN, LF power, and HF power were analyzed after log transformation due to non‐normal distribution. Comparisons between groups for quantitative data were performed using Student's test if normally distributed and signed rank test (Mann–Whitey) if not. Statistical analysis and graph were performed with GraphPad Prism (RRID:SCR_002798). A p value below α = 0.05 was considered significant.

## 3 RESULTS

From September 2018 to January 2020, 21 patients with type 1 diabetes met the selection criteria, including availability of PSG data (premature interruption due to the SARS‐CoV‐2 pandemic lockdown). We divided the population as planned: 14 patients had AHI < 15/h and 7 patients ≥15/h. Descriptive and comparisons of baseline characteristics of the study groups are shown in Table 1. Compared to patients with AHI < 15/h, patients with an AHI ≥ 15/h had a significantly higher AHI during REM sleep (39.4 ± 15.8 vs. 12.9 ± 9.2; *p* < 0.01), as well as during non‐REM sleep (27.6 ± 16.1 vs. 5.4 ± 2.8; *p* < 0.01).

**TABLE 1:** Patients' characteristics and comparison of all T1D patients and T1D patients with AHI< or ≥15/h.

	All T1D patients	T1D patients	T1D patients	*p* Value
		AHI < 15/h	AHI ≥ 15/h	
	(*n* = 21)	(*n* = 14)	(*n* = 7)	
Age (years)	46 ± 13	42 ± 11	52 ± 16	0.10
Male	11 (57%)	6 (43%)	5 (71%)	0.36
BMI (kg/m^2^)	26.2 ± 4.2	25.8 ± 3.8	27.1 ± 5.1	0.51
Diabetes duration (years)	27 ± 13	26 ± 11	30 ± 16	0.43
HbA1c (%)	8.5 ± 1.2	8.9 ± 1.3	7.8 ± 0.6	**0.05**
Basal‐bolus injections (%)	9 (47)	6 (43)	3 (43)	1.00
Insulin pump (%)	12 (63)	8 (57)	4 (57)	1.00
≥1 cardiovascular disease	1 (5%)	0 (0%)	1 (14%)	0.16
Chronic kidney disease	5 (24%)	4 (29%)	1 (14%)	0.52
Retinopathy	11 (52%)	9 (64%)	2 (28%)	0.14
Peripheric neuropathy	3 (14%)	2 (14%)	1 (14%)	0.94
Autonomic neuropathy	2 (10%)	2 (14%)	0 (0%)	0.31
AHI (/h)	16 ± 15	7.8 ± 3.5	33.4 ± 15.2	**<0.01**
AHI REM (/h)	21.7 ± 17.3	12.9 ± 9.2	39.4 ± 15.8	**<0.01**
AHI NREM (/h)	12.8 ± 14.2	5.4 ± 2.8	27.6 ± 16.1	**<0.01**
ODI (/h)	10 ± 14	3.4 ± 3.5	23.6 ± 17.5	**0.02**
Time spent under 90% SaO2	3 ± 5	0.9 ± 1.8	6.8 ± 7.5	0.09
Total sleep time (min)	362 ± 51	366 ± 47	353 ± 62	0.47
Sleep efficiency (%)	72 ± 11	73 ± 10	71 ± 14	0.22
Arousal index (/h)	19.8 ± 8.0	16.7 ± 7.5	25.9 ± 5.2	**0.01**
WASO (min)	97 ± 69	90 ± 58	110 ± 91	0.29
N1 (%)	6 ± 3	5 ± 2	8 ± 3	**0.02**
N2 (%)	60 ± 8	59 ± 6	62 ± 10	0.13
N3 (%)	15 ± 6	16 ± 6	14 ± 7	0.29
REM (%)	19 ± 6	20 ± 4	16 ± 7	0.08
PLM (/h)	21.0 ± 29.1	21.6 ± 35.8	19.8 ± 6.8	0.74
PLM with arousals (/h)	3.3 ± 4.1	3.6 ± 4.8	2.6 ± 2.2	0.62

*Note*: Values are expressed as means ± standard deviations or number (percentage). Comparisons by two‐tailed unpaired Student's *t*‐test or two‐sided Chi‐square test. Bold values indicate statistically significant differences (*p* < 0.005).

Abbreviations: AHI, apnea‐hypopnea index; BMI, body mass index; N2, sleep 2 stage; N3, sleep 3 stage; ODI, oxygen desaturation index; PLM, periodic limb movements; REM, rapid eye movement sleep stage; T1D, type 1 diabetes; WASO, wake after sleep onset.

### 3.1 Heart rate variability

**FIGURE 1:**
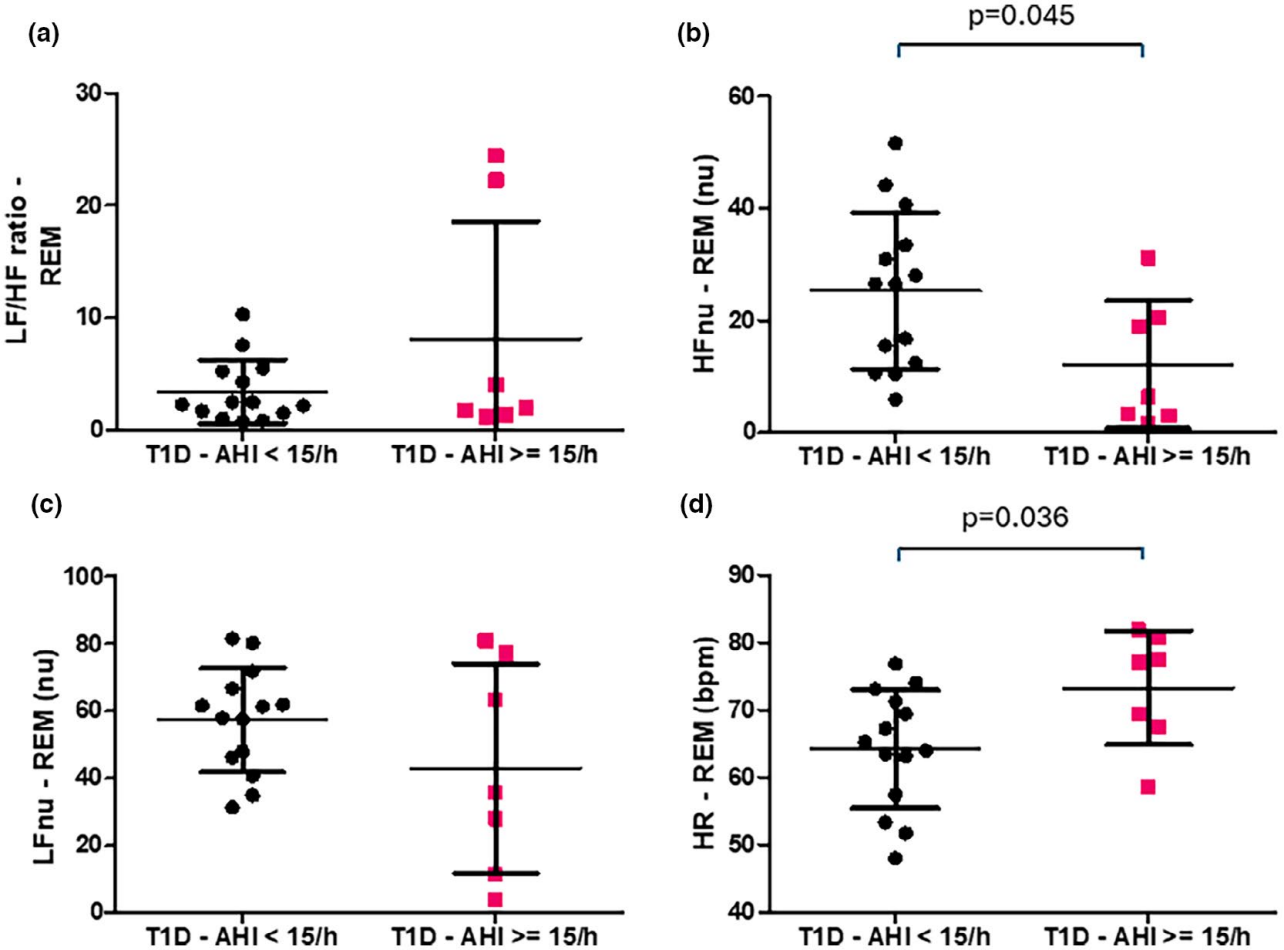
Comparison of HRV parameters between T1D patients with AHI< (black, *n* = 14) or ≥15/h (pink, *n* = 7) for (a) LF/HF ratio in REM, (b) HFnu in REM, (c) LFnu in REM, (d) HR in REM. Values are expressed as means ± standard deviation. AHI, apnea‐hypopnea index; HFnu, high‐frequency band power in normalized units; HR, heart rate; LF/HF ratio, low‐frequency band power/high‐frequency band power ratio; LFnu, low‐frequency band power in normalized units; REM, rapid eye movement sleep stage.

Compared to T1D patients with AHI < 15/h, T1D patients with AHI ≥ 15/h showed no significant difference in LF/HF ratio in N2, N3 or REM sleep stages (see Appendix S1). However, LF/HF ratio in REM was positively correlated with AHI (*r* = 0.44; *p* = 0.05) across all patients. When comparing other HRV parameters, a significantly lower HFnu in REM was observed in T1D patients with AHI ≥ 15/h versus patients with AHI < 15/h (12.1 ± 11.4 vs. 25.3 ± 14.0 nu; *p* < 0.05, Figure 1) and was accompanied by a higher HR in REM (73 ± 8 vs. 64 ± 9 bpm; *p* < 0.05, Figure 1). Last, in our whole population of T1D patients HRV analyses during sleep confirmed that the REM sleep was more prone autonomic nervous system disturbance than N3 sleep stage. Indeed, SDNN and LF/HF ratio were significantly higher in REM versus N3 sleep stages (51.7 ± 18.8 vs. 28.8 ± 13.0 ms; *p* < 0.01 and 5.0 ± 4.3 vs. 1.2 ± 0.6; p < 0.01, respectively, Appendix S1). In addition, HFnu was significantly lower in REM versus N3 sleep stage (20.9 ± 11.9 vs. 46.3 ± 12.1 nu; *p* < 0.01, Appendix S1).

### 3.2 Cardiovascular reflex tests, sudomotor dysfunction and catecholamines

In our study population, 12 (57.1%) patients had no CAN, regardless of AHI. Two (14.3%) patients had a possible CAN, and 6 (28.6%) patients had a confirmed or severe CAN, including 3 patients with AHI ≥ 15/h. We found a positive correlation between the decrease in systolic blood pressure during postural change and AHI (*r* = 0.58; *p* = 0.01). Using Sudoscan®, one patient with T1D and AHI < 15/h and one patient with T1D and AHI ≥ 15/h presented with sudomotor dysfunction affecting both hands and feet. Both patients presented with severe CAN according to CARTs.

Regarding catecholamine measurements, we found a significantly higher NE/E ratio and a trend toward higher urinary norepinephrine adjusted to creatinine levels in T1D patients with AHI ≥ 15/h in comparison with those with AHI < 15/h (Table 2).

**TABLE 2:** Urinary catecholamines in T1D patients with AHI< or ≥15/h.

	T1D patients	T1D patients	*p* Value
	AHI < 15/h	AHI ≥ 15/h	
	(*n* = 14)	(*n* = 7)	
Norepinephrine/creat (μmol/mol)	11.82 ± 7.60	20.85 ± 15.90	0.09
Epinephrine/creat (μmol/mol)	2.42 ± 1.20	2.02 ± 1.48	0.52
NE/E ratio	3.45 ± 3.01	16.22 ± 17.04	**0.03**
Normetanephrine/creat (mmol/mol)	0.10 ± 0.03	0.12 ± 0.05	0.22
Metanephrine/creat (mmol/mol)	0.05 ± 0.03	0.04 ± 0.01	0.27

*Note*: Values are expressed as means ± standard deviations. Comparisons by two‐tailed unpaired Student's *t*‐test. Bold values indicate statistically significant differences (*p* < 0.05).

Abbreviations: AHI, apnea‐hypopnea index; creat, creatinine; NE/E ratio, norepinephrine/epinephrine ratio.

## 4 DISCUSSION

To our knowledge, this is the first study that reports the impact of OSA on HRV and CAN in patients with type 1 diabetes. An AHI ≥ 15/h was associated with significant alterations in HRV indices with a lower HFnu in REM, accompanied by a higher HR in REM, indicating a diminished parasympathetic activity in the presence of OSA. In addition, the NE/E ratio, a marker of the peripheral sympathetic nervous system activity, was significantly higher in patients with an AHI ≥ 15/h.

Previous studies have reported a deleterious effect of OSA on the sympathovagal balance during sleep in non‐diabetic patients. Yet, in contrast with our hypothesis, we did not find a significant higher LF/HF ratio during each sleep stage in T1D patients with AHI ≥ 15/h. Nevertheless, and in line with previous studies (Park et al., 2008), we found a statistically significant correlation between LF/HF in REM and AHI, meaning that including a larger sample of OSA patients with AHI ≥ 15/h could have increased the LF/HF ratio in this group. In addition, in our T1D patients, there was a higher urinary NE/E ratio in patients with AHI ≥ 15/h, which is consistent with an increase in the catecholamine release by the peripheral sympathetic system (Robertson et al., 1979; Yamaguchi et al., 1988). Altogether, our results in T1D patients are in favor of higher sympathetic activity during sleep in T1D patients with AHI ≥ 15/h.

Besides the sympathetic activation during REM sleep, T1D patients with an AHI ≥ 15/h showed also a lower HFnu and a higher HR during REM sleep in comparison with T1D patients with an AHI < 15/h, in favor of a decreased parasympathetic activity related to OSA during REM stage. Previously, in patients without T1D, Guilleminault et al. have also reported a decrease in HF indicating a significant drop in parasympathetic tone after the respiratory events (Guilleminault et al., 2005). A recent systematic review and meta‐analysis in patients with OSA has confirmed that the reduction in HF and impairment of markers of parasympathetic function during sleep in OSA patients (Wang et al., 2023).

Despite evidence of a sympatho‐vagal imbalance during sleep, we did not find any diurnal autonomic nervous system dysregulation. Indeed, diurnal CARTs as well as wakefulness HRV did not reveal any significant difference between patients with AHI< and ≥15/h. Yet, this was limited by the low number of patients with confirmed or severe CAN. Interestingly, Palma et al. also failed to observe any abnormal HRV profile during wakefulness in OSA patients with AHI ≥ 15/h, while their HRV profile during sleep was impaired versus healthy control subjects (Palma et al., 2015). In fact, there is a discrepant literature regarding the respective impairment of diurnal or nocturnal indices of HRV in OSA. Recently, Qin et al. observed that the HRV impairment during wakefulness (5 min sample before PSG) was restricted to the most severe OSA patients (Qin et al., 2021). In addition, Nam et al. have shown that the diurnal HRV was less impaired than the nocturnal HRV in OSA (Nam et al., 2022). Altogether, we could hypothesize that diurnal HRV alterations may be seen in the most severe OSA patients. This hypothesis is supported by the correlation between orthostatic hypotension—a marker of autonomic nervous system dysregulation—and AHI across all patients, which became statistically significant with the most severe patients (*r* = 0.58, *p* = 0.01, Figure S1). Thus, given that only one third of our T1D patients showed a moderate to severe OSA syndrome, our population would not be severe enough to show significant diurnal HRV impairment.

Currently, there is an increasing concern regarding the deleterious cardiovascular impact of OSA in diabetes patients. Obstructive sleep apnea is not rare in T1D patients, representing 16.7% in a meta‐analysis (Reutrakul & Mokhlesi, 2017), and one in four diabetic patients with autonomic neuropathy (Ficker et al., 1998). It remains frequently underdiagnosed because patients do not show increased daytime sleepiness (Banghoej et al., 2017). In T1D patients, OSA exerts a cumulative effect on CVD (Valensi et al., 2025). Diabetes and OSA exert a bidirectional interplay between cardiovascular disorders (Riley et al., 2025; Yang et al., 2022). While CAN paves the way for CVD in patients with T1D (Astrup et al., 2006), the autonomic nervous system dysregulation observed during sleep in our T1D patients with an AHI ≥ 15/h could promote CAN and CVD in T1D patients, as evidenced in animal models (Brooks et al., 1997) and patients (Carlson et al., 1993; Taylor et al., 2016). Recently, functional MRI studies have evidenced the structural and functional basis of chronic autonomic nervous system dysfunction and CAN in OSA (Fatouleh et al., 2014; Lin et al., 2020; Taylor et al., 2018). Thus, a dysregulation of the autonomic nervous system during sleep should be considered as a threat in T1D patients. Due to the retrospective constitution of the study groups and the limited study sample, we acknowledge potential study limitations. In our T1D patients with AHI ≥ 15/h, they tended to be older than patients with an AHI < 15/h. Since HRV is known to decline with age, this may have influenced our findings. However, in a meta‐analysis including 172 studies (63,612 participants), the effect of age was weak and not significant regarding the HFnu (Koenig & Thayer, 2016). Conversely, HbA1c levels were significantly higher in T1D patients with AHI < 15/h. While glycemic control assessed by HbA1c has been associated with worse diurnal HRV indices in T1D (Hajdu et al., 2023; Jaiswal et al., 2013), this higher HbA1c level in patients may have masked larger effects of OSA nocturnal HRV and CAN. Last, despite significant differences observed in HRV during sleep between groups, the small sample size and unbalanced study groups (*n* = 7, *n* = 14) constitute a study limitation. Altogether, despite its cross‐sectional design, our study provides a multi‐faceted assessment of the autonomic nervous system dysfunction in T1D patients, with consistent results regarding HRV measurements and CAN in T1D patients.

## 5 CONCLUSION

Despite its cross‐sectional design, the multi‐faced assessment of the autonomic nervous system dysfunction in patients with T1D showed consistent results indicating the deleterious effect of OSA on the nocturnal autonomic nervous system. Given that cardiovascular prevention constitutes a major issue in patients with T1D, future longitudinal studies are warranted to determine whether OSA exacerbates CAN independently of glycemic control. Furthermore, investigating sympathetic responses to respiratory events and the hypoxic burden according to CAN severity could provide mechanistic insights into the amplified cardiovascular risk observed in patients with T1D and OSA.

## AUTHOR CONTRIBUTIONS

**Marion Faivre:** Data curation; formal analysis; funding acquisition; investigation. **Fares Gouzi:** Data curation; formal analysis; funding acquisition; investigation; supervision. **Jérôme Thireau:** Data curation; formal analysis; funding acquisition; investigation; software; supervision. **Bronia Ayoub:** Data curation; formal analysis; funding acquisition; investigation. **Justine Myzia:** Data curation; formal analysis; funding acquisition; investigation. **Orianne Villard:** Data curation; formal analysis; funding acquisition; investigation. **Amandine Suc:** Data curation; formal analysis; funding acquisition; investigation. **Maurice Hayot:** Conceptualization; formal analysis; funding acquisition; investigation. **François Roubille:** Conceptualization; formal analysis; funding acquisition; investigation. **Yves Dauvilliers:** Conceptualization; formal analysis; funding acquisition; investigation. **François Bughin:** Conceptualization; data curation; formal analysis; funding acquisition; investigation; methodology; software.

## FUNDING INFORMATION

The study was partially funded by SOS oxygène and Groupe Adène companies.

## CONFLICT OF INTEREST STATEMENT

No conflicts of interest, financial or otherwise, are declared by the authors.

## ETHICS STATEMENT

The study was approved by review boards (CPP IDF1 n°2017‐A03438‐45), conducted in accordance with Helsinki's declaration.

## Supporting information


Appendix S1.

## Data Availability

Source data for this study are not publicly available due to privacy or ethical restrictions. The source data are available to verified researchers upon request by contacting the corresponding author.
