# Supplementary material for: Deleterious impact of obstructive sleep apnea on autonomic nervous system control during rapid‐eye‐movement sleep in adult type 1 diabetes
Source: Physiol Rep. 2026 Jun 16;14(12):e70951. doi: 10.14814/phy2.70951 (PMC13273019; doi:10.14814/phy2.70951)
Supplement: Supplementary file 1 — Appendix S1. [file PHY2-14-e70951-s001.docx]

**Supplementary material: Deleterious impact of obstructive sleep apnea on autonomic nervous system control during rapid-eye-movement sleep in adult type 1 diabetes**

**Methods**

*Polysomnography*

The parameters related to sleep architecture were the following: total sleep time, time spent in bed, wake after sleep onset, N1 sleep, N2 sleep, N3 sleep, REM sleep, sleep efficiency, and arousal. The arousal index (AI) was determined as the total number of arousals per hour. Periodic limb movement was measured (PLM) such as those associated with arousals.

The oxygen desaturation index was determined by counting the number of events in which oxygen saturation decreased by 3% or more per hour. Apnea was characterized as a reduction in airflow ≥90% of baseline for a minimum duration of 10 s. Hypopnea was characterized by a reduction in airflow ≥30% of baseline for a minimum duration of 10 s, accompanied by either an arousal or a 3% decrease in oxygen saturation. The apnea-hypopnea index (AHI) was determined by the number of apnea-hypopnea events per hour of recorded sleep time. Patients were classified into non or mild OSA (AHI < 15), and moderate or severe OSA (AHI ≥ 15) groups.

*Cardiovascular autonomic reflex tests (CARTs)*

Heart rate and blood pressure responses were assessed during 5 standardized maneuvers. (Chowdhury et al. 2021)

A/ Heart rate response to deep breathing: Patients are asked to breathe at a rate of six times per minute, with approximately 5s of inhalation and 5s exhalation per breath. We calculated the expiratory-inspiratory ratio (E:I ratio) which is the ratio of the longest RR interval during expiration divided by the shortest RR interval during inspiration from 5 cycles. Values below 1.2 are considered abnormal results.

B/Heart rate response to standing. Patients are asked to rest in the supine position for a specified amount of time, then to stand up. We calculate the ratio between the longest RR interval (around the 30^th^ heartbeat) and the shortest RR interval (around the 15^th^ heartbeat) after posture change. Values below 1.04 are considered abnormal results.

C/ Valsalva maneuver: During expiration, patients are asked to maintain a mercury column at 40 mmHg for 15s. This leads to tachycardia, and after 30-45s bradycardia. We calculate the ratio between the shortest RR interval divided by the longest RR interval. Values below 1.21 are considered abnormal results.

D/ Blood pressure response to standing up: Also called orthostatic hypotension test, it explores variations in blood pressure between the rest period and after standing for three min. The decrease in systolic blood pressure and/or diastolic blood pressure should be < or = 20 and/or 10 mmHg, respectively.

E/ Blood pressure response to sustained handgrip: The isometric handgrip test consists of pressing the handgrip with nearly 30% of the maximum contraction strength for 3-5 min. This maneuver could be performed with dominant arm and/or the non-dominant arm and is supposed to promote an increase in diastolic blood pressure. Blood pressure is measured in the contralateral arm and an increase of at least 15 mmHg between the rest and peak effort values is expected.

**Supplementary Table 1.** Heart Rate Variability measures and comparison of T1D patients with AHI < or ≥ 15/h

|  | **T1D patients AHI < 15/h** | **T1D patients AHI ≥ 15/h** | **p-value** |
| --- | --- | --- | --- |
| **RR_W (ms)** | 897.5±131.0 | 817.7±55.0 | 0.11 |
| **HR_W(bpm)** | 68.4±9.8 | 74.1±5.1 | 0.24 |
| **TP_W (ms²)** | 1833.2±2111.7 | 2157.4±2852.6 | 0.84 |
| **SDNN_W (ms)** | 45.1±28.3 | 50.9±44.0 | 0.73 |
| **RMSSD_W** | 23.6±13.1 | 21.0±17.2 | 0.73 |
| **LF_W (ms²)** | 488.8±569.8 | 565.7±880.7 | 0.97 |
| **HF_W (ms²)** | 168.1±142.8 | 296.4±371.2 | 0.27 |
| **LFnu_W (nu)** | 56.3±14.9 | 46.4±33.5 | 0.37 |
| **HFnu_W (nu)** | 29.3±14.8 | 29.4±20.7 | 0.98 |
| **LF/HF ratio_W** | 2.9±2.6 | 3.6±3.4 | 0.25 |
| **RR_N2 (ms)** | 940.3±141.5 | 898.5±100.7 | 0.42 |
| **HR_N2 (bpm)** | 65.2±9.3 | 67.6±7.5 | 0.62 |
| **TP_N2 (ms²)** | 923.3±763.3 | 1632.0±2807.0 | 0.40 |
| **SDNN_N2 (ms)** | 32.1±15.4 | 34.4±27.6 | 0.98 |
| **RMSSD_N2 (ms)** | 26.4±17.6 | 26.2±32.2 | 0.92 |
| **LF_N2 (ms²)** | 357.5±326.5 | 504.9±946.1 | 0.48 |
| **HF_N2 (ms²)** | 232.4±282.4 | 356.2±735.6 | 0.52 |
| **LFnu_N2 (nu)** | 54.5±20.5 | 61.4±9.1 | 0.42 |
| **HFnu_N2 (nu)** | 39.5±19.2 | 32.3±10.9 | 0.21 |
| **LF/HF ratio N2** | 2.0±1.8 | 2.2±1.2 | 0.55 |
| **RR_N3 (ms)** | 955.3±146.9 | 923.3±84.6 | 0.23 |
| **HR_N3 (bpm)** | 64.2±9.2 | 65.5±5.6 | 0.75 |
| **TP_N3 (ms²)** | 856.2±1406.6 | 1490.2±2689.4 | 0.40 |
| **SDNN_N3 (ms)** | 27.8±17.7 | 31.1±27.6 | 0.75 |
| **RMSSD_N3 (ms)** | 28.6±23.9 | 29.5±34.0 | 0.95 |
| **LF_N3 (ms²)** | 275.2±498.7 | 482.6±869.9 | 0.45 |
| **HF_N3 (ms²)** | 244.9±333.5 | 508.2±1039.9 | 0.29 |
| **LFnu_N3 (nu)** | 45.0±14.3 | 49.5±17.0 | 0.41 |
| **HFnu_N3 (nu)** | 48.3±13.8 | 41.9±14.5 | 0.36 |
| **LF/HF ratio N3** | 1.1±0.6 | 1.5±1.0 | 0.89 |
| **RR_REM (ms)** | 955.4±142.1 | 833.3±104.8 | 0.47 |
| **HR_REM (bpm)** | 64.2±8.8 | 73.2±8.4 | 0.04 |
| **TP_REM (ms²)** | 2871.0±3004.7 | 5103.7±7356.8 | 0.33 |
| **SDNN_REM (ms)** | 49.9±21.5 | 55.3±24.9 | 0.94 |
| **RMSSD_REM (ms)** | 31.2±20.2 | 21.5±13.6 | 0.31 |
| **LF_REM (ms²)** | 607.5±636.4 | 796.8±1260.8 | 0.65 |
| **HF_REM (ms²)** | 257.5±315.5 | 148.6±202.3 | 0.56 |
| **LFnu_REM (nu)** | 57.3±15.5 | 42.9±31.2 | 0.29 |
| **HFnu_REM (nu)** | 25.3±14.0 | 12.1±11.4 | 0.04 |
| **LF/HF ratio REM** | 3.5±2.8 | 8.2±10.4 | 0.29 |

Data are presented as means ± standard deviations or number (percentage). Comparisons by two-tailed unpaired Student’s *t* test. Abbreviations: T1D, type 1 diabetes; AHI, apnea-hypopnea index; N2, stage 2 sleep; N3 stage 3 sleep; REM, rapid eye movement sleep stage; V, wake; RR, R-R intervals; HR, heart rate; TP, total power; SDNN, standard deviation of normal-to-normal R-R intervals; RMSSD, root mean square of successive differences between normal-to-normal R-R intervals; HF, high-frequency power; LF, low-frequency power; HFnu, high-frequency band power in normalized units; LFnu, low-frequency band power in normalized units; LF/HF ratio, low-frequency band power/high-frequency band power ratio;

**Supplementary Table 2.** Cardiovascular reflex tests and comparison of T1D patients with AHI < or ≥ 15/h

|  | **T1D patients**  **AHI < 15/h** | **T1D patients**  **AHI ≥ 15/h** | **p-value** |
| --- | --- | --- | --- |
| **Deep breathing - RR ratio** | 1.3±0.2 | 1.3±0.2 | 0.95 |
| **Deep breathing - abnormal test (n)** | 3 (21%) | 2 (29%) | 0.60 |
| **Lying to standing - RR ratio** | 1.3±0.2 | 1.2±0.2 | 0.32 |
| **Lying to standing - abnormal test (n)** | 1 (7%) | 0 (0%) | 1.00 |
| **Valsalva maneuver - ratio RR** | 1.4±0.2 | 1.5±0.4 | 0.29 |
| **Valsalva maneuver - abnormal test (n)** | 3 (21%) | 2 (29%) | 0.60 |
| **Systolic BP response to standing (mmHg)** | 10.3±6.7 | 15.2±11.0 | 0.24 |
| **Systolic BP response to standing - abnormal test (n)** | 2 (15%) | 2 (29%) | 0.59 |
| **Diastolic BP response to handgrip (mmHg)** | 24.4±9.2 | 21.7±8.7 | 0.55 |
| **Diastolic BP response to handgrip - abnormal test (n)** | 1 (8%) | 0 (0%) | 1.00 |
| **No CAN** | 9 (64%) | 3 (50%) | 1.00 |
| **Possible CAN** | 2 (14%) | 0 (0%) | 1.00 |
| **Confirmed CAN** | 2 (14%) | 3 (50%) | 0.61 |
| **Severe CAN** | 1 (7%) | 0 (0%) | 1.00 |

Data are presented as means ± standard deviations or number (percentage). Comparisons by two-tailed unpaired Student’s *t* test or two-sided Chi-square test. Abbreviations: T1D, type 1 diabetes; AHI, apnea-hypopnea index; RR, R-R intervals; BP, blood pressure; CAN, cardiac autonomic neuropathy

**Supplementary figure 1**. Correlation analysis

Correlations between HTO and AHI across all patients (r=0.58, p=0.01). Abbreviations: AHI, apnea-hypopnea index; HTO, absolute difference in systolic blood pressure during postural change.

**Supplementary figure 2**. Comparison of HRV parameters across all patients between REM et N3 sleep stages.


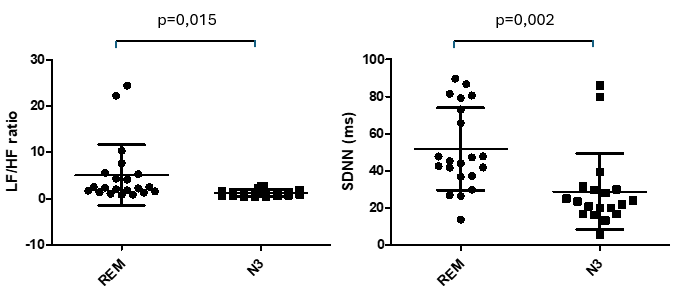


Values are expressed as means ± standard deviations. Comparisons by two-tailed unpaired Student’s *t* test. Abbreviations: LF/HF ratio, low-frequency band power/high-frequency band power ratio; SDNN, standard deviation of normal-to-normal R-R intervals; REM, rapid eye movement sleep stage; N3, slow-wave sleep stage.
